# Effect of laser pulse energy and repair composite viscosity on the repair shear bond strength of aged bulk-fill resin composites

**DOI:** 10.3389/fdmed.2026.1795256

**Published:** 2026-03-23

**Authors:** Nesrine A. Elsahn, Maan Ahmad Alshouli, Saleh Aneess Bahdar, Muhammad Sohail Zafar

**Affiliations:** 1 Clinical Sciences Department, College of Dentistry, Ajman University, Ajman, United Arab Emirates; 2 Centre of Medical and Bio-allied Health Sciences Research, Ajman University, Ajman, United Arab Emirates; 3 Department of Conservative Dentistry, Faculty of Dentistry, Cairo University, Cairo, Egypt; 4 Postgraduate Program in Prosthodontics, College of Dentistry, Ajman University, Ajman, United Arab Emirates; 5 Private Practitioner, Alfarabi Medical Center, Ajman, United Arab Emirates; 6 School of Dentistry, University of Jordan, Amman, Jordan

**Keywords:** air abrasion, bond strength, bulk-fill composite, ER:YAG laser, repair, roughness, surface topography

## Abstract

**Background:**

Repairing failed resin composite restorations remains clinically challenging, and the longevity of repaired restorations depends on achieving a strong interfacial bond. This study evaluated the influence of different surface treatments and repair composite types on the repair shear bond strength (RBS) of aged bulk-fill resin composites.

**Materials and methods:**

One hundred and fifteen standardized Filtek™ One Bulk-Fill Restorative (3M) composite discs (4 mm × 10 mm) were thermocycled (5,000 cycles, 5–55 °C). Specimens were divided into five surface treatment groups: Er:YAG laser at 50 mJ (L50), 150 mJ (L150), and 250 mJ (L250), air abrasion (A), and diamond bur control (B). After surface treatment, a universal adhesive was applied, and three composite cylinders (2 mm × 2 mm) were built per specimen using either Filtek Z250 XT Nano-Hybrid (N), Filtek Bulk-Fill Flowable (F), or Filtek One Bulk-Fill Restorative (R) (*n* = 10). Repaired samples were thermocycled again (×5,000) and tested for RBS. Surface roughness (Ra, Sa) was assessed by profilometry and AFM, and SEM micrographs were used to examine surface morphology. Data were analyzed using two-way and one-way ANOVA with *post hoc* tests (*α* = 0.05).

**Results:**

Surface treatment and its interaction with the repair composite type significantly influenced RBS (*p* < 0.05), whereas the repair composite type did not significantly influence RBS (*p* > 0.05). The highest RBS values were recorded for L250 and Air Abrasion, while L50 yielded the lowest across all composites. L250 showed the highest Ra and Sa values. AFM and SEM confirmed pronounced peaks, valleys, and microretentive pits in the L250 and Air Abrasion groups, whereas smoother surfaces were observed following bur and low-energy laser treatments.

**Conclusions:**

High-energy Er:YAG laser (250 mJ) and air abrasion significantly enhanced repair bond strength by generating retentive surface topographies. The repair composite type had minimal influence, though low-viscosity materials performed better on highly ablated surfaces due to improved flow and adaptation.

## 1 Introduction

Despite advances in the formulation of resin-based composites (RBCs), clinical failures such as discoloration, microleakage, marginal ditching, wear, or fracture remain common—often due to environmental degradation in the oral cavity (1, 2). Complete replacement of a failed restoration is no longer the gold standard, since it may lead to additional loss of sound tooth structure, increased risk of pulpal insult, and greater treatment costs and complexity. Consequently, repair procedures have emerged as a conservative, minimally invasive approach that increases the longevity of restorations, cost-effectiveness, and preserves dental tissues. For a successful composite repair, achieving reliable interfacial bonding between aged and fresh composite layers is essential (3). This is typically accomplished using intermediate bonding agents and/or surface treatments (4). Low-viscosity composites, silane coupling agents, and adhesives, particularly those containing functional monomers such as 10-methacryloyloxydecyl dihydrogen phosphate (MDP), have been proposed to improve adhesion by interacting with the residual resin matrix of aged composites, and promoting chemical bonding (5–7). A variety of surface pretreatment strategies have been investigated to enhance repair bond strength (RBS), including abrasion with diamond burs, acid etching, air abrasion with aluminum oxide or silica-coated particles, and laser-assisted surface treatment (4). These surface treatments aim to increase surface energy and surface roughness, thereby enhancing micromechanical retention and adhesion between the aged and new composite materials (8).

Recently, erbium-doped yttrium aluminum garnet (Er:YAG) lasers have demonstrated the ability to precisely and selectively ablate composite surfaces with minimal thermal damage and maximal preservation of healthy tooth structure, while simultaneously creating an ideal micro-retentive surface (9) via a hydrokinetic ablation process. These characteristics, which contrast with the induced thermal damage or reduced bonding potential observed with other laser systems, make the Er:YAG laser a promising and effective tool for treating failed restoration surfaces, with the potential to improve the longevity and success of repaired dental restorations (10). The Er:YAG laser energy is highly absorbed by water and hydroxyl groups within the composite's resin matrix. Rapid heating leads to the vaporization of these components, creating high internal pressure that causes “micro-explosions” (11). However, the efficacy of laser irradiation is closely linked to its interaction with the ablated surface, which is governed by several factors, including the absorption and scattering coefficients of the substrate, as well as laser parameters such as pulse energy, duration, repetition rate, and the use of water cooling (12). Optimizing these variables for each substrate is essential to achieving predictable and clinically successful outcomes. Although the efficacy of air abrasion in enhancing bond strength to repaired composite restorations has been extensively validated in previous studies, there remains a scarcity of investigations examining the use of Er:YAG and Er,Cr:YSGG lasers for repairing aged resin composites (13–15). However, the effectiveness of these lasers as surface treatments remains controversial, with studies reporting inconsistent or contradictory outcomes compared with conventional approaches such as air abrasion or bur preparation (10, 15). Moreover, the use of non-standardized, highly variable laser settings in these studies renders direct comparisons of their outcomes unreliable (13, 14). To date, no standardized protocol for laser parameters in resin composite repair has been established, and the influence of specific laser settings on the repair bond strength (RBS) of restorations has not been thoroughly investigated.

Simultaneously, the advent of bulk-fill resin composites has revolutionized restorative protocols, allowing for 4–5 mm increments to be placed and cured efficiently while minimizing polymerization shrinkage, owing to their unique chemistry (16, 17). A systematic review and meta-analysis reported a failure rate of 5.57% for bulk-fill resins over a follow-up period of 12–72 months, compared with 3.32% for conventional incrementally layered resin composites (18). These similar clinical outcomes between the two composite types were further confirmed in a recent systematic review, which evaluated follow-up intervals ranging from 6 months to 10 years (19), reinforcing the growing popularity of bulk-fill composites. The use of an adhesive without any prior mechanical surface treatment is not recommended for bulk-fill resin repairs (20). Despite this recommendation, evidence comparing the influence of mechanical surface alteration methods on the repairability of aged bulk-fill composites remains limited, with most available research focusing on the effects of bur roughening or air abrasion as surface treatments for bulk-fill resin composite repairs (21–24). Although Er,Cr:YSGG laser irradiation has been explored in a single study (25). The influence of varying laser settings and the compatibility between laser-ablated aged bulk-fill surfaces and different composite types or viscosities have yet to be examined.

Therefore, this *in vitro* study aimed to evaluate the effect of different Er:YAG laser settings and surface treatments on the shear bond strength (SBS) of three different resin composite materials used to repair thermocyclic-aged bulk-fill composites. The tested null hypotheses were:

1. Laser pulse energy and surface treatment technique do not significantly affect the bond strength of repaired bulk-fill resin composites.
2. The type of repair composite has no significant influence on the repair bond strength.

## 2 Materials and methods

### 2.1 Preparation and aging of specimens

The present *in vitro* study was conducted in accordance with the Declaration of Helsinki. Table 1 describes the details of all the resin composite materials (Filtek™ One Bulk Fill, Filtek™ Z250 XT Nano Hybrid, Filtek™ Bulk Fill Flowable) used in this study. A total of 115 cylindrical resin composite specimens (4 mm × 10 mm) were fabricated using Filtek™ One Bulk Fill Restorative composite, shade A3 (3M ORAL CARE), according to the manufacturer's guidelines. Briefly, preformed silicone molds were placed over a transparent Mylar matrix strip (Universal Strip, DML, Germany), which had been previously aligned on a clean glass slide. The bulk-fill composite was dispensed into the molds using a composite dispenser until the molds were filled. To ensure surface smoothness and uniform packing and to eliminate the oxygen-inhibited layer, a second mylar strip, a glass slide, and a standardized weight of 250 g were applied to the top surface. After removing the weight and any excess material, each specimen was light-cured in two overlapping 20 s cycles using a standard curing unit (Litex 696 LED Cordless Curing Light, Dentamerica, CA, USA), with the curing tip positioned perpendicularly and in direct contact with the Mylar strip.

**Table 1:** Materials used in the study.

Material brand name	Manufacturer	Chemical composition	lot number
Filtek™ one bulk fill restoratives	3M Oral Care, St. Paul, MN, USA	AFM, AUDMA, UDMA, and 1,12-dodecane-DMA. Non-agglomerated/non-aggregated 20 nm silica filler; 4–11 nm zirconia filler; aggregated zirconia/silica cluster filler (20 nm silica and 4–11 nm zirconia particles); ytterbium trifluoride filler (agglomerated 100 nm particles). Inorganic filler 76.5% by weight (58.4% by volume).	NA46725
Filtek™ Z250 XT Nano Hybrid Universal Restorative	3M Oral Care, St. Paul, MN, USA	BIS-GMA, UDMA, BIS-EMA, PEGDMA, and TEGDMA; surface-modified zirconia/silica (median particle size ≤3 μm); 20 nm surface-modified silica; filler loading 82% by weight (68% by volume).	N968941
Filtek™ Bulk Fill Flowable Restorative	3M Oral Care, St. Paul, MN, USA	BisGMA, UDMA, bisEMA and Procrylat resins; zirconia/silica 0.01–3.5 μm and ytterbium trifluoride 0.1–5.0 μm; inorganic filler 64.5% by weight (42.5% by volume).	N952645
Single Bond Universal Adhesive	3M Oral Care, St. Paul, MN, USA	MDP Phosphate Monomer, Dimethacrylate resins, HEMA, Vitrebond Copolymer, Filler, Ethanol, Water, Initiators, Silane.	B4135911

AFM, additional fragmentation monomer; Bis-GMA, bisphenol A-glycidyl methacrylate; UDMA, urethane dimethacrylate; Bis-EMA, ethoxylated bisphenol A dimethacrylate; TEGDMA, triethylene glycol dimethacrylate; AUDMA, aromatic urethane dimethacrylate; PEGDMA, polyethylene glycol dimethacrylate; MDP, 10-methacryloyloxydecyl dihydrogen phosphate; HEMA, 2-hydroxyethyl methacrylate.

The unit emitted light at approximately 1,200 mW/cm^2^ in the 400–500 nm wavelength range, and its output was periodically verified throughout the study using a calibrated radiometer. Cured samples were embedded in dental stone (WhipMix, Louisville, KY, USA) within cylindrical acrylic molds, exposing only the top composite surface. The exposed surfaces were polished using 1,200-grit silicon carbide abrasive paper for 15 s under running water with a grinder-polisher device (Buehler, USA), thereby standardizing surface roughness. All specimens underwent thermocycling between 5 °C and 55 °C with 30 s dwell times for 5,000 cycles to simulate oral thermal aging (THE-1200 Thermocycler, SD Mechatronik GmbH, Germany).

### 2.2 Surface treatment protocols

After aging, the specimens were randomly divided into five surface treatment groups (*n* = 23 per group), using a computer-generated random number table (Table 2).

**Table 2:** Variables of the study and study groups.

Surface treatment	Filtek™ one bulk fill restoratives (bulk restorative, R)	Filtek™ Z250 XT nano hybrid universal restorative (nano-hybrid, N)	Filtek™ bulk fill flowable restorative (bulk flow, F)
Er:YAG at 50 mJ (L50)	RL50	NL50	FL50
Er:YAG at 150 mJ (L150)	RL150	NL150	FL150
Er:YAG at 250 mJ (L250)	RL250	NL250	FL250
Air abrasion (A)	RA	NA	FA
Bur preparation (B)	RB	NB	FB

Group 1 (L50): Er:YAG laser irradiation at 1 W power (50 mJ/pulse)

Group 2 (L150): Er:YAG laser at 3 W (150 mJ/pulse)

Group 3 (L250): Er:YAG laser at 5 W (250 mJ/pulse)

Group 4 (Air Abrasion): Sandblasting with 50 µm aluminum oxide particles for 10 s at 60 PSI and a 5 mm working distance, using an intraoral sandblaster (Microetcher II, Danville Engineering Inc., USA)

Group 5 (Bur): Surface roughening with a diamond bur (8837KR.314.012, Komet, Germany). The bur was replaced after every five specimens.

For groups 1, 2, and 3, laser surface conditioning was performed using an Er:YAG laser system (Fidelis, Fotona Medical Lasers, Slovenia) in focused mode at a fixed distance of 2 mm using a custom apparatus. The beam (0.9 mm spot size) was delivered with a non-contact handpiece (R02) in sweeping motion, with a pulse duration of 100 μs (MSP mode) at 20 Hz for 10 s, under a water-air spray (3% water, 6% air). After treatment, all specimens were thoroughly rinsed and air-dried.

### 2.3 Shear bond strength (SBS) testing

Sample size was determined based on shear bond strength data reported by Ayar et al. (26), using G*Power software (Version 3.1). Assuming *α* = 0.05 and power (1−*β*) = 0.80, a minimum of 8 specimens per group was required. To increase statistical robustness and account for potential specimen loss during preparation or testing, 10 specimens were randomly allocated to each of the 15 experimental subgroups. For SBS testing, each pre-treated surface received two consecutive layers of Single Bond Universal Adhesive (3M ORAL CARE), gently agitated for 20 s per layer, followed by air-drying for 5 s, and then light-cured for 20 s using the same curing device. Subsequently, three composite repair materials (Filtek™ One Bulk Fill Restorative, Filtek™ Z250 XT Nano Hybrid Universal Restorative, and Filtek™ Bulk Fill Flowable Restorative) were built over each pretreated surface using prefabricated (2 mm × 2 mm) polypropylene cylindrical molds (Ultradent, USA). The composite was packed and then cured for 20 s, following the manufacturer's instructions. This procedure was repeated three times per specimen, using a different composite each time. The top of each cylinder was marked with a color-coded permanent marker for identification.

All specimens were stored in distilled water at 37 °C for 24 h, followed by a second thermocycling phase (5,000 cycles) under the same parameters before mechanical testing. Shear bond strength testing was conducted using a universal testing machine (M350-5CT, Testomatric, UK). A shear force was applied at the adhesive interface at a crosshead speed of 0.5 mm/min until failure. Bond strength values were calculated in MPa by dividing the recorded failure load (N) by the bonded area (mm^2^).

### 2.4 Surface roughness evaluation

#### 2.4.1 Atomic force microscopy (AFM)

After surface treatment, five additional specimens from each group were assigned for surface roughness analysis using an Atomic Force Microscope (AFM) (Flex-Axiom, Nanosurf AG, Switzerland). Four random rectangular areas (25 µm × 25 µm each) per sample were scanned in non-contact mode using a cantilever tip (NCLR: 7 µm thickness, 225 µm length, 38 µm width) under a standard force of 48 N/m. The AFM data were analyzed using C3000 control software (v3.7.2.8). The average surface roughness (Sa) was recorded in nanometers (nm) and then converted to micrometers (µm).

#### 2.4.2 Profilometry

Surface roughness was also assessed using a surface profilometer (Surftest-211, Mitutoyo Corp., Japan) equipped with a 2-μm diamond stylus, operating at 0.5 mm/s with a cutoff of 0.25 mm. The stylus traversed 4 mm per measurement, and three readings per specimen were averaged to determine Ra values for each of the five samples per group.

### 2.5 Micro-topographical examination (SEM)

Three additional samples per group were prepared for Scanning Electron Microscopy (SEM). After surface treatment, specimens were washed, air-dried for 60 s, and stored in a desiccator with silica gel for 24 h. The dried surfaces were sputter-coated with gold-palladium (Polaron E-5100, UK) at 20 mA for 90 s. Samples were examined using a low-vacuum SEM (JSM 5310LV, JEOL Inc., Japan) at an accelerating voltage of 20 kV, with a working distance of 12–17 mm. Micrographs were taken at magnifications up to 2,000×.

The investigator conducting SBS testing and SEM/AFM analyses was blinded to the surface treatment groups to reduce bias.

### 2.6 Statistical analysis

Data were expressed as means ± standard deviations (SD). SBS values were analyzed using a Two-way ANOVA to evaluate the main effects and interaction between surface treatment and repair composite type. One-way ANOVA was applied for surface roughness parameters, followed by a Bonferroni *post-hoc* test for pairwise comparisons. Statistical significance was set at a *p*-value of ≤0.05. All analyses were performed using SPSS software v16.0® (IBM, USA).

## 3 Results

**Figure 1:**
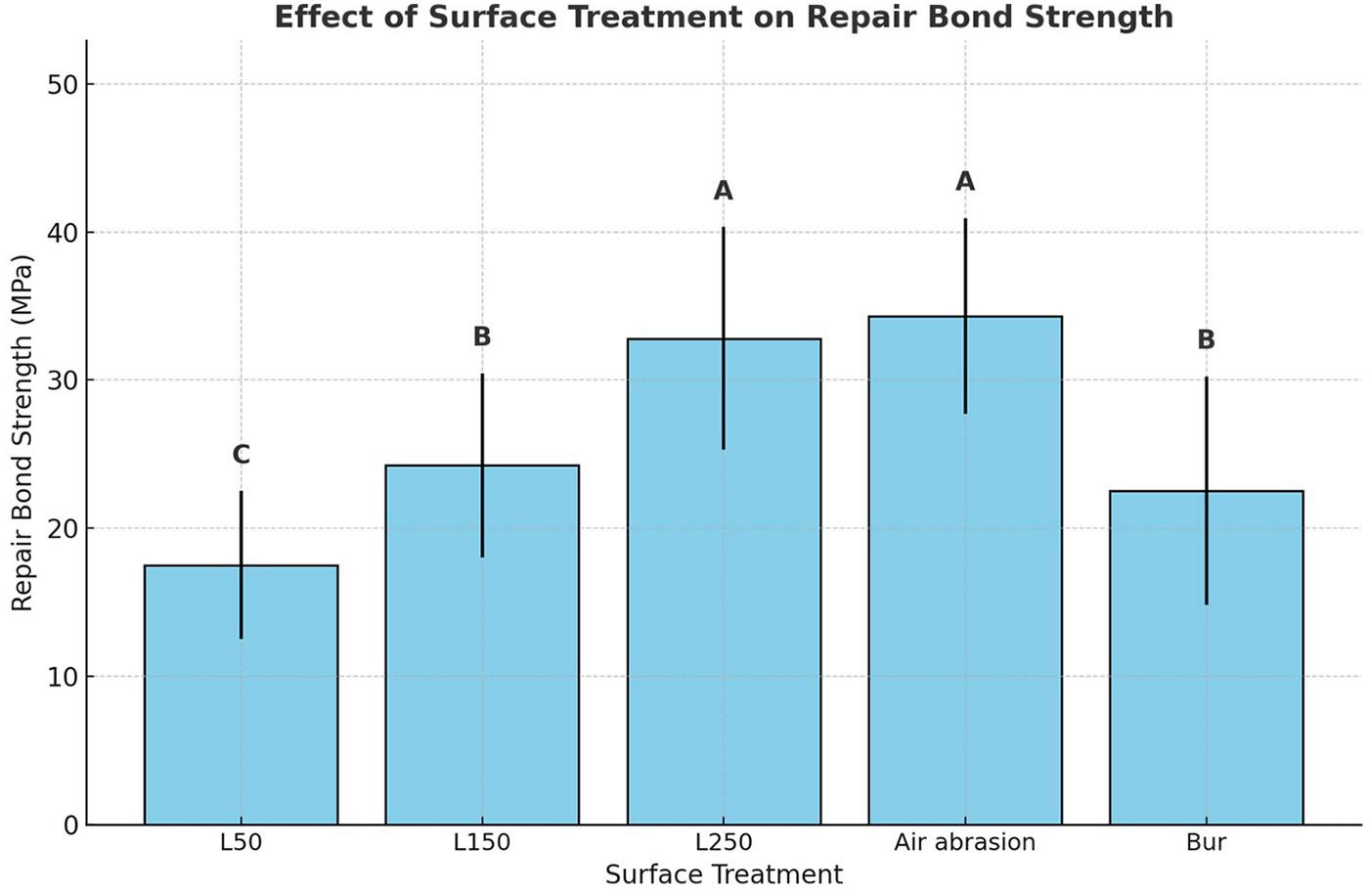
Effect of surface treatment on the repair bond strength. Bars represent mean bond strength (MPa); different uppercase letters indicate statistically significant differences between groups (*p* < 0.05).

**Figure 2:**
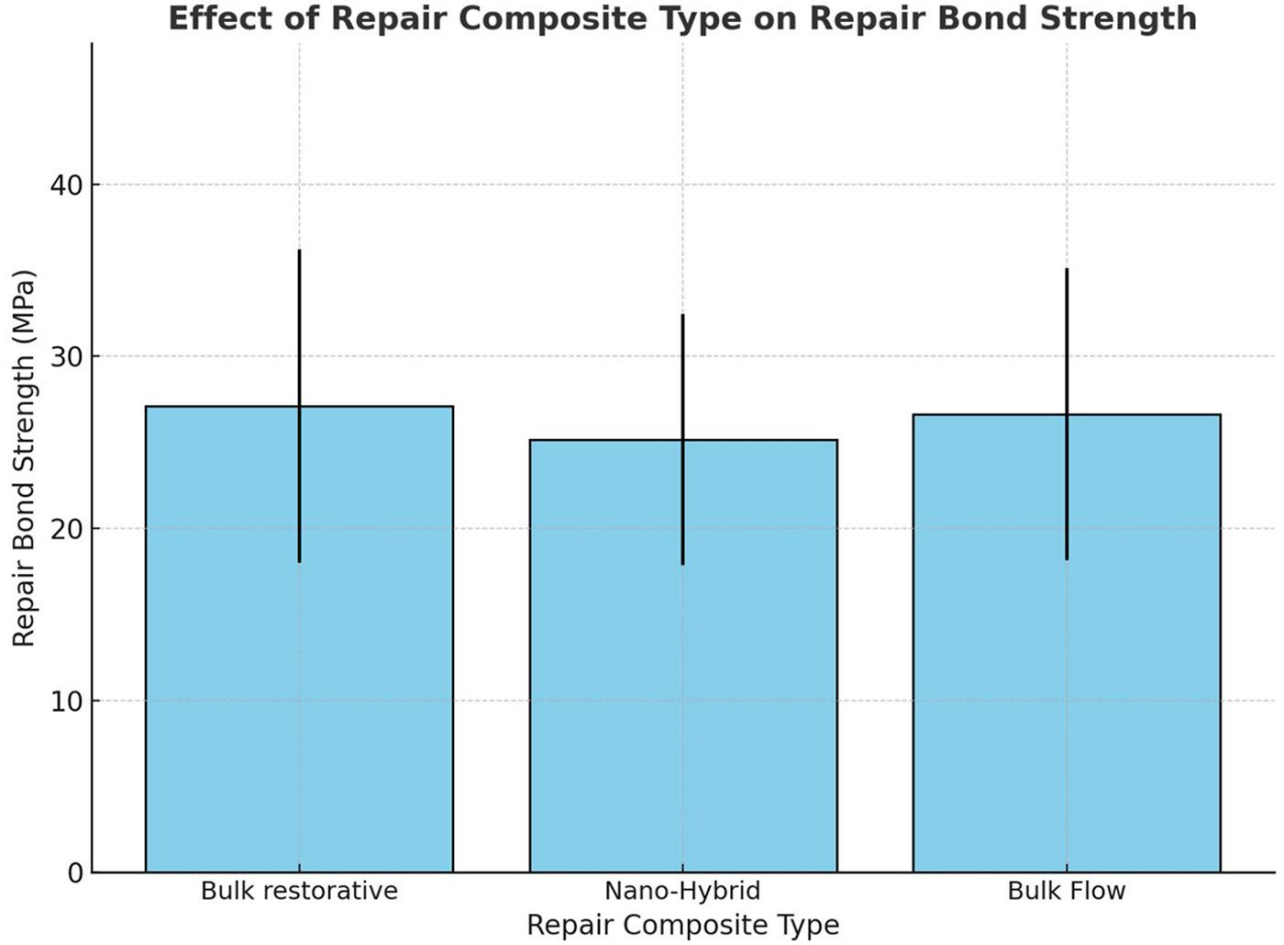
Mean repair bond strength (MPa) for each repair composite type (bulk restorative, nano-hybrid, and bulk flow). Bars represent the mean values, with no statistically significant difference between the groups (*p* > 0.05).

Two-way ANOVA revealed that surface treatment had a statistically significant influence on repair shear bond strength (*p* < 0.05). As illustrated in Figure 1, the highest mean bond strength was recorded for the air abrasion and L250 groups (34.33 MPa and 32.82 MPa, respectively), which were statistically similar. On the other hand, the lowest value was found for the L50 group (17.53 MPa). No statistically significant difference in SBS was found between the L150 and bur Groups (*p* > 0.05). Figure 2 shows the effect of repair composite type on bond strength. The mean bond strength values for Bulk Restorative, Nano-Hybrid, and Bulk Flow composites were 27.10 MPa, 25.16 MPa, and 26.62 MPa, respectively. Statistical analysis revealed that the type of repair composite material didn't significantly influence the RBS (*p* > 0.05). However, the interaction between surface treatment and composite type produced a statistically significant effect on RBS.

The repair shear bond strength (RBS) values across all combinations of surface treatments and repair composites are presented in Table 3. Pairwise comparisons revealed that only within the L250 groups did the RBS vary significantly by repair composite type. The highest RBS was recorded for RL250 (40.5 MPa ± 6.9 MPa), followed by FL250 (32.8 MPa ± 3.3 MPa), while the lowest was observed in NL250 (25.2 MPa ± 2.7 MPa). Overall, the strongest bonds were obtained with L250 and air abrasion treatments, whereas the weakest bonds occurred consistently in the L50 groups across all composite types. No statistically significant differences in RBS were observed between the L250 and air-abrasion groups when bulk-fill materials were used for repair; notably, the NA group exhibited a significantly higher RBS than the NL250 group.

**Table 3:** Repair shear bond strength of the tested groups (MPa).

Surface treatment	Filtek™ one bulk fill restoratives	Filtek™ Z250 XT nano hybrid universal restorative	Filtek™ bulk fill flowable restorative
L50	14.9 ± 2.3 Da	17.5 ± 5.8 Ba	20.1 ± 6.1 Ba
L150	20.9 ± 5.7 CDa	23.0 ± 6.4 Ba	28.9 ± 9.2 ABa
L250	40.5 ± 6.9 Aa	25.2 ± 2.7 Bc	32.8 ± 3.3 Ab
Air Abrasion	33.6 ± 7.5 ABa	35.0 ± 3.5 Aa	33.5 ± 9.3 Aa
Bur	25.7 ± 4.2 BCa	24.1 ± 11.8 Ba	21.2 ± 6.3 Ba

Within each column, mean values with different uppercase letters are statistically significantly different (*p* < 0.05). Different lowercase letters indicate significant differences within each row (*p* < 0.05).

Table 4 shows the mean surface roughness measured using the profilometer (Ra) and atomic force microscopy (SA). Among the tested groups, L250 produced the highest surface roughness (Ra = 13.57 µm ± 1.35 µm; SA = 2.29 µm ± 0.45 µm), which was significantly higher than that of the other groups (*p* < 0.05). The air abrasion and bur groups showed the lowest roughness values, with no significant differences between them, while L50 and L150 exhibited intermediate levels.

**Table 4:** Surface roughness mean (standard deviation) of the tested groups.

Surface treatment	Ra (Profilometer, µm)	SA (AFM, µm)
L50	6.63 (1.10)^b^	1.29 (0.30)^b^
L150	9.42 (0.47)^c^	1.11 (0.28)^b^
L250	13.57 (1.35)^d^	2.29 (0.45)^c^
Air Abrasion	2.90 (0.51)^a^	0.733 (0.098)^a^
Bur	3.73 (0.42)^a^	0.726 (0.12)^a^

Different lowercase letters in the same column indicate statistically significant differences (*p* < 0.05).

**Figure 3:**
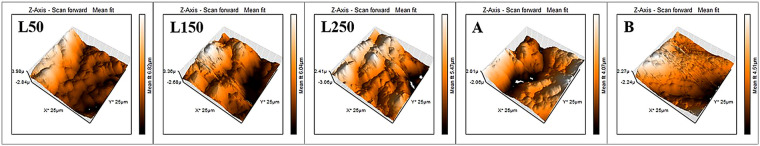
Three-dimensional atomic force microscopy images of the tested surface treatment groups: Er:YAG laser at 50 mJ (L50), 150 mJ (L150), 250 mJ (L250), Air abrasion **(A)**, and Bur preparation **(B)** the images reveal variations in surface roughness and topography created by different treatments.

**Figure 4:**
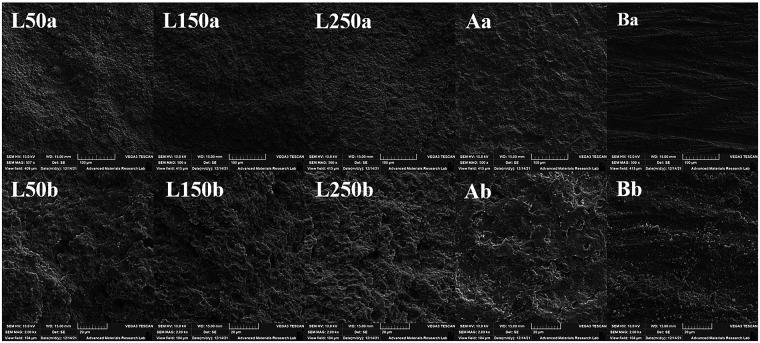
Scanning electron microscopy (SEM) micrographs of the tested surface treatment groups at two magnifications: **(a)** ×500 and **(b)** ×2,000. Er:YAG laser at 50 mJ (L50a, L50b), 150 mJ (L150a, L150b), 250 mJ (L250a, L250b), Air Abrasion (Aa, Ab), and Bur Preparation (Ba, Bb). The images illustrate the surface morphology and micro-roughness induced by each treatment.

Atomic force microscopy (Figure 3) and scanning electron microscopy (Figure 4) revealed distinct differences in surface topography among the tested surface treatments. The L250 group exhibited the highest and most irregular peaks and valleys, corresponding to its significantly higher roughness values, as reported in Table 4. Moreover, under SEM, it displayed a micro-roughened surface with deep fissures and irregularities. Air abrasion produced a moderately rough surface with localized peaks, as shown in the AFM image, and yielded a uniformly pitted surface under SEM. In contrast, bur preparation resulted in smoother contours, flatter striations, and fewer undercuts. The L50 and L150 groups showed intermediate roughness and relatively shallow surface features, with the L50 group being the least textured of the laser-treated specimens.

## 4 Discussion

The current study investigated the influence of different surface treatments on the repair shear bond strength (RBS) of aged bulk-fill resin composites and further characterized them using a surface profilometer, AFM and SEM. The results demonstrated that a high-energy Er:YAG laser (250 mJ) and air abrasion significantly enhanced repair bond strength by generating retentive surface topographies. Therefore, the first null hypothesis was rejected. To preserve sound tooth structure, repair of a defective resin composite restoration is now considered a more conservative and clinically advantageous option than complete replacement (3). However, direct bonding between aged and fresh composites has historically yielded suboptimal results due to the absence of the oxygen-inhibited layer of unpolymerized monomers, which is crucial for chemical bonding between composite layers on aged surfaces, as well as compromised integrity of the filler–resin matrix interface resulting from material degradation (27). Over time, composite restorations undergo various forms of degradation, including physical, chemical, and hydrolytic, which lead to the leaching of unreacted monomers and soluble ions, and to the breakdown of interfacial bonds within the material; however, bulk-fill materials exhibit the highest stability among incrementally packed composites tested (28, 29).

To simulate intraoral degradation, thermocycling was applied twice in the present study: first to artificially age the composite substrate specimens, and second to evaluate the repair bond's durability. This process exposed the samples to alternating thermal extremes, mimicking the stresses encountered in the oral cavity during daily functions such as eating and drinking. The adopted thermocycling regimen corresponded to approximately six months of clinical service, a commonly accepted standard for evaluating the effects of artificial aging on dental materials (30, 31). This protocol provided a realistic simulation of both thermal and hydrolytic degradation of the composite substrate, enabling a meaningful assessment of the bonding performance between the aged and newly applied repair layers under conditions that closely replicate the oral environment (29).

Previous studies have consistently shown that aging and thermocycling adversely affect the repair bond strength of both conventional incrementally layered and bulk-fill composites (16). Eren and Çobanoglu (32) also reported that immediate repairs with bulk-fill composites yielded clinically acceptable RBS values, whereas delayed repairs exhibited significantly lower strength. On the other hand, mechanical surface treatment using a bur or sandblasting reduced the effect of aging on the repair bond strength (RBS) of bulk-fill material compared with repair performed only with an adhesive, confirming that mechanical surface modification is necessary to restore the bonding potential of aged composites (20). Another study concluded that the detrimental effect of 5,000 cycles of thermocycling on RBS was more pronounced in specimens treated with bur preparation, while it had no significant influence on samples treated with laser irradiation (33). This finding suggests that laser-treated surfaces may demonstrate greater resistance to thermal degradation, possibly due to their more stable micro-retentive morphology and the superior integrity of the adhesive interface.

The present study demonstrated that surface treatment plays a crucial role in achieving durable adhesion between aged and freshly applied composite layers. Er: YAG laser irradiation at 250 mJ (L250) and air abrasion achieved the highest RBS values, whereas the low-energy laser group (L50) consistently produced the weakest bonds across all repair composite types (Figure 1 and Table 3). These findings confirm the pivotal role of mechanical surface modification in establishing durable composite repairs and highlight that the effectiveness of different surface treatments and laser energies varies significantly. Previous research has shown that the use of adhesives without mechanical pretreatment is not recommended, particularly for bulk-fill composite repair, as mechanical surface preparation is essential to achieve sufficient micromechanical retention and bonding performance (16, 20, 23). Mechanical surface conditioning removes surface contaminants such as plaque, debris, and the degraded outer composite layer that develops through water sorption and aging. This process exposes a fresh, more reactive substrate, thereby increasing surface energy and improving the adhesive's penetration and wettability. In addition, it enhances micromechanical interlocking, particularly when methods such as air abrasion or high-energy laser treatment are employed.

Air abrasion uses a high-velocity stream of aluminum oxide (Al_2_O_3_) particles to generate a uniformly roughened surface with numerous micro-irregularities, as confirmed by SEM analysis in the current study (Figure 4). Laser ablation modifies the surface via explosive vaporization and hydrodynamic ejection (11), creating microcraters and pores that become increasingly pronounced with higher laser energy (Figures 3, 4). This was accompanied by a measurable increase in surface roughness (Table 4). Such surface features enlarge the effective bonding area and permit the adhesive resin to flow into these microporosities, forming a strong mechanical interlock with the newly exposed reactive surface. These factors explain the significantly higher RBS observed in the air-abraded and L250 groups after thermocycling. The superior RBS of these two groups may also be attributed to their ability to clean the composite surface effectively, thereby altering its surface energy and wettability, thus enhancing adhesion and adhesive penetration. These improvements facilitate stronger chemical adhesion and copolymerization with the repair composite, producing a more cohesive and durable hybrid layer between the old and new materials. Consistent with earlier study, laser ablation was found to be as effective as air abrasion in preparing aged composite surfaces for repair (34, 35). The high bond strength observed in the air-abrasion groups also aligns with previous reports showing that alumina particle abrasion, when combined with appropriate adhesive systems, with or without an additional silane step, serves as one of the most effective surface treatments for composite repair. It yields greater bond strength compared with no preparation, phosphoric acid etching, or diamond bur treatment (22, 23).

Similarly, in the present study, L250 group results are consistent with those of Celiksoz et al. (11), who found that Er:YAG laser irradiation at 5 W significantly improved the RBS of aged nano-hybrid composites compared to bur-treated groups after 5,000 thermocycles. The superior performance of the air-abraded and L250 groups compared to the bur-treated group in this study aligns with Kimyai et al. (35), who found that an Er:YAG laser at 2 W achieved RBS values higher than bur preparation, but their study did not include aging procedures. The absence of an aging phase and the possible presence of an oxygen-inhibited layer in their samples may have artificially enhanced laser group performance, even at low energy levels. Further supporting evidence was provided by Rashidi et al. (34), who reported that an Er:YAG laser at 3 W and 300 mJ on aged micro-hybrid composites produced RBS values similar to those of air abrasion, and both were significantly higher than those of bur-treated or untreated controls. Likewise, Hasan (36) observed that low-energy Er:YAG irradiation (40–60 mJ) increased the immediate RBS of aged nanocomposites compared with air-abraded and bur-treated samples after 300 thermocycles. In that study, however, phosphoric acid etching and the use of a flowable intermediate layer may have enhanced bonding and contributed to the high reported values, even at low-laser energy.

In the present study, Er:YAG lasers at 1 W and 3 W produced RBS values statistically similar to those of the bur-treated groups, except in the RL50 subgroup, where the RBS was significantly lower. Previous studies have reported similar findings (13, 15, 33), who found no significant differences between laser-ablated and bur-treated composites, regardless of laser settings or aging conditions. Conversely, Kiomarsi et al. (37), reported that Er,Cr:YSGG laser ablation at 3 W was less effective than bur treatment, while Barcellos et al. (38), observed that applying laser treatment after bur preparation actually reduced RBS, suggesting potential over-roughening or surface damage.

Regardless of the repair composite used, air abrasion consistently outperformed low-energy laser groups (L50 and L150). This result agrees with previous studies (14, 39) who found that air abrasion achieved higher RBS than Er,Cr:YSGG laser at 5 W on nano-hybrid composites and Er:YAG at 4 W on micro-hybrid composites, respectively—though both studies measured RBS without post-repair aging. These collective findings reinforce that mechanical pretreatment remains indispensable for achieving reliable and durable adhesion in composite repair procedures, and that high-energy Er:YAG laser irradiation may serve as a viable, minimally invasive alternative to air abrasion when properly optimized.

The interaction between laser irradiation and composite substrates is complex and influenced by multiple variables, including laser type, operating parameters, and substrate composition. Different laser types vary in wavelength, energy-absorption characteristics, and ablation mechanisms, which account for variations in their surface effects. For instance, Oskoee et al. (40), reported that the Er,Cr:YSGG laser was more effective than Nd:YAG and CO₂ lasers for treating silorane-based composites, highlighting that specific laser systems produce more favorable surface morphologies, whereas others may induce thermal damage or reduced bonding potential. In addition to the laser type, application parameters—including pulse energy, power output, duration, repetition rate, and water cooling—directly influence the extent of surface modification and the resultant micromechanical properties. The material composition of the ablated composite, as well as the adhesive system and repair composite subsequently applied, also affect the final RBS. Evidence consistently demonstrates that laser parameters significantly affect the repair bond strength (RBS). Although higher power settings result in deeper ablation and greater surface roughness, excessive energy can produce heat-induced surface damage—including melting, microcracking, and irregular crater formation—that may compromise micromechanical retention (41). Such surface distortion can diminish the adhesive's ability to infiltrate the substrate, reducing effective interfacial bonding.

Duran et al. (42), confirmed that both air abrasion and Er:YAG laser treatment significantly increased the immediate RBS of aged micro-hybrid composites, achieving values above the clinical threshold proposed by Teixeira et al. (43). However, their findings differed from the present study, the highest RBS was observed at 75 mJ, whereas the lowest was at 300 mJ. The authors attributed this reduction at higher power settings to the formation of a thermally damaged, loosely attached superficial layer on the subsurface composite, as well as to excessive ablation that reduced the effective bonding area. The lower laser energies might preserve the integrity of the substrate surface, preventing deterioration and maintaining better adhesion. Similarly, a reduction in RBS for aged nano-filled composites after 300 thermocycles was reported when pulse energy was increased from 60 to 80 mJ (36). However, their methodology involved applying laser irradiation at only five discrete spots (1 mm apart), which may have limited overall surface modification. In contrast, other studies have suggested that 300 mJ pulse energy is optimal for achieving reliable surface roughness and bonding in ceramics and denture resins, whereas both higher and lower energies were associated with decreased bond strength (44).

Conversely, Batista et al. (45), concluded that although increasing laser energy produced a rougher surface, it did not significantly affect the tensile RBS. Similarly, Rossato et al. (46), reported that raising the pulse energy from 200 to 300 to 400 mJ did not significantly alter the shear bond strength. In both studies, the RBS values in the laser-treated groups were statistically comparable to those achieved with air abrasion or bur preparation. It should be noted, however, that none of these studies subjected their repaired samples to aging, and most employed different post-treatment cleaning protocols, such as ultrasonic cleaning or phosphoric acid etching, which may have contributed to the variability of their results. Collectively, these findings highlight that laser–substrate interaction is multifactorial and that both excessive and insufficient energy can compromise repair outcomes. Establishing standardized parameters, considering laser type, energy settings, and cleaning protocols, is therefore essential for achieving reproducible and clinically reliable surface conditioning in composite repair.

One of the main clinical challenges in restoration repair is that the type of failed composite substrate cannot always be identified, particularly when the repair is performed by a clinician other than the one who placed the original restoration. This variability is clinically significant because the effectiveness of erbium lasers may depend on the composition and filler characteristics of the composite material being treated (34). Several studies have demonstrated that laser–substrate interaction varies according to the composite formulation. For instance, Ghavam et al. (10), and Rashidi et al. (34), found that Er,Cr:YSGG laser irradiation reduced bond strength in giomer composites, while it improved adhesion in microhybrid and nanohybrid composites. Similarly, Karaarslan et al. (47) reported that Er:YAG laser irradiation at 1.2 W was particularly effective for nanofilled composites, achieving repair bond strengths comparable to those with air abrasion, regardless of the adhesive or repair composite used. However, for nanohybrid composites, the laser's efficiency was more dependent on the specific adhesive system and repair materials. Furthermore, Rashidi et al. (34) suggested that composites with higher resin content, such as microhybrids, may develop a thicker, more viscous superficial layer after irradiation, which resists effective cleaning and etching, thereby impairing subsequent bonding. Condensable composites exhibited less material removal compared with microfilled and hybrid types, while Kiomarsi et al. (37), associated the ease of ablation and potential for surface damage with the lower cohesive strength of specific composite formulations. These findings underscore that the composition of the composite critically determines how efficiently and safely a laser can modify its surface for optimal bonding.

Despite the growing body of literature on laser surface treatment of various composite types, only one study by Atalay et al. (25) has investigated the effect of Er, Cr:YSGG laser irradiation at 1.5 W specifically on bulk-fill composites. Their results showed that both laser conditioning and aluminum oxide (Al₂O₃) sandblasting produced the highest bond-strength values compared with untreated or acid-etched surfaces. The superior performance of high-energy Er:YAG laser treatment and air abrasion observed in the present study is consistent with those findings. In contrast, Atalay et al. (25), employed a microtensile bond strength test, which is known to induce higher stress concentrations at the interface and generally yield lower RBS values for bulk-fill composites (48). This distinction may explain why the Er:YAG laser at up to 3 W in our study produced RBS values lower than those of the air-abraded groups. Moreover, the two studies differed in their aging protocols: Atalay et al. (25) measured bond strength after 24 h of storage, while our specimens underwent 5,000 thermal cycles before testing. Thermocycling likely deteriorated the initially formed bonds, yet the durability of the high-energy laser-treated interfaces remained comparable to that of the air-abraded groups. This finding emphasizes a critical consideration: aging of repaired samples substantially affects the measured RBS values. Studies that omit post-repair aging may report artificially elevated bond strengths that do not reflect long-term clinical behavior. Thus, incorporating realistic aging simulations is essential for accurately assessing the durability and reliability of composite repair bonds.

The surface roughness results (Table 4) closely mirrored the repair bond strength (RBS) outcomes. Complementary three-dimensional topographic imaging by atomic force microscopy (AFM) (Figure 3) and surface morphology observed in scanning electron microscopy (SEM) micrographs at ×500 (a) and ×2,000 (b) magnifications (Figure 4) provided consistent visual evidence of these findings. Among the tested groups, the L250 treatment exhibited the highest Ra and Sa values, characterized by pronounced peaks and valleys in AFM imaging, indicating intense micro-ablation and a highly retentive surface—findings consistent with those of Cho et al. (39) Air abrasion, while producing moderately rough surfaces, achieved bond strengths comparable to those of other methods, likely due to the combined effects of micro-retention and enhanced surface cleanliness. Under SEM examination, both L250 and air-abrasion specimens exhibited deep fissures, exposed filler particles, and irregular pits, all of which are conducive to strong micromechanical interlocking, corroborating the observations of Ahmadizenouz et al. (49). According to Eren et al. (50), surface topography exerts a more pronounced effect on RBS than surface roughness alone, and Batista et al. (45) further noted that no direct linear correlation always exists between roughness values and bond strength.

In contrast, lower-energy laser treatments (L50 and L150) and bur preparation produced significantly lower Ra and Sa values than the L250 group, resulting in smoother striations and fewer undercuts. These less retentive surfaces were associated with correspondingly reduced RBS values. L50 setting likely provided only superficial cleaning without adequate ablation depth to create micromechanical retention, limiting adhesive infiltration and bonding efficacy. The diminished performance of bur-treated specimens can be attributed to the presence of a smear layer. This weakly attached surface film lowers surface energy and impedes wetting and chemical bonding with the repair resin. While bur preparation typically produces macro-retentive surface irregularities, laser irradiation, particularly at higher energy levels, generates a combination of micro- and macro-retentive features that promote superior micromechanical interlocking (35).

Consistent with Şişmanoğlu et al. (21), who reported a strong positive correlation between surface roughness and RBS (*r* = 0.831), the present study also found that within the laser-treated groups, RBS increased proportionally with both pulse energy and roughness parameters (Ra and Sa). Specifically, L150 exhibited intermediate roughness and bond strength, whereas L50 showed the lowest roughness and RBS, displaying the least textured surface among all laser groups. Laser ablation of resin composites selectively removes the polymeric matrix, exposing and partially releasing the filler particles (49). Materials with lower cohesive strength or weaker filler–matrix bonds tend to be more susceptible to ablation, leading to more pronounced microstructural alterations (49). In the present study, low-energy laser treatment did not induce sufficient ablation to create a retentive surface on the tested bulk-fill material, whereas intermediate-energy (L150) treatment achieved surface characteristics comparable to those of bur treatment. However, high-energy Er:YAG irradiation (L250) proved most effective in modifying the surface of Filtek™ One Bulk Fill Restorative, producing a well-textured, microretentive morphology that supported durable bonding between aged and newly applied composites. This effect was particularly evident when a low-viscosity repair composite was used, which readily penetrated surface micro-irregularities, enhancing interfacial adaptation and long-term repair stability.

Consistent with the ANOVA results, no significant differences were observed among the Bulk Restorative, Nano-Hybrid, and Bulk Flow repair composites when standardized repair protocols were applied, regardless of the surface treatment used. Therefore, the second null hypothesis was accepted. This finding supports previous research demonstrating that aged bulk-fill composites can be effectively repaired with either conventional nanohybrid or microhybrid composites, provided that adequate surface conditioning is performed (23, 26, 51). Conversely, in the absence of mechanical surface treatment, pretreatment with a silane coupling agent followed by a hydrophobic adhesive and the use of the same bulk-fill material for repair have been recommended to enhance the bond strength of bulk-fill restorations (6). Our findings, however, differ from those of Benzi and Oglakci (52), who reported superior bond strength when aged bulk-fill composites were repaired using the same material—Filtek™ One Bulk Fill and Tetric EvoCeram Bulk Fill, respectively. During composite repair, the bond between the new and aged composite relies on both chemical and micromechanical interactions. Chemical adhesion occurs through bonding of the organic matrix or exposed fillers (after silanization), while micromechanical retention increases surface energy and bonding surface area by exposing a fresh, reactive substrate. The combination of these mechanisms ensures a stronger and more durable interface between the two composite layers.

Although bulk-fill composites differ in their monomeric composition and polymer network, the material used in this study contains AFM (Addition–Fragmentation Monomer) and AUDMA (Aromatic Urethane Dimethacrylate), which may, in theory, limit chemical bonding with conventional composites. Nevertheless, in agreement with our results, de Medeiros et al. (17) reported high compatibility between bulk-fill and conventional composites when appropriate adhesive protocols were followed. It has been postulated that unreacted AFM monomers exposed during surface treatment can fragment and repolymerize under lower-stress conditions, thereby forming new cross-links with the adhesive system, an effect that may enhance chemical bonding, as suggested by the manufacturer (52). Interestingly, in the present study, a significant interaction between surface treatment and repair material type was observed only in the L250 group, in which the aged bulk-fill composite repaired with bulk-fill material exhibited higher RBS values than those restored with conventional composites. This outcome is likely due to the lower viscosity of bulk-fill composites, which facilitates their flow into surface irregularities created by high-energy laser ablation, thereby improving wettability and micromechanical interlocking. Similarly, Hasan (36) who found higher RBS values on laser-ablated surfaces than on air-abraded or bur-treated specimens when an intermediate flowable composite layer was used.

According to Teixeira et al. (42), RBS values ranging between 15 and 25 MPa are considered clinically acceptable for durable adhesion. In the present study, all surface treatments, regardless of the repair material used, achieved bond strengths within or above this range, confirming the effectiveness of the evaluated repair strategies in restoring the integrity of aged bulk-fill composites. This *in vitro* study is limited by its controlled laboratory conditions, including controlled conditions that do not fully replicate the oral environment. The absence of clinical aging factors such as biofilm, saliva enzymes, and occlusal stress cannot be fully simulated through thermocycling alone. In addition, the study's limitations include the use of a single adhesive system and a bulk-fill material, as well as a restricted range of laser parameters. Future clinical studies with more extended follow-up periods and a broader evaluation of laser settings and adhesive–composite combinations are recommended to establish standardized protocols for laser-assisted composite repair.

## 5 Conclusions

The present findings reinforce that surface treatment plays a more dominant role than composite type in determining the effectiveness of bulk-fill composite repair. Once optimal micromechanical retention is achieved, the selection between bulk-fill, flowable, or nano-hybrid composites can be guided primarily by clinical convenience, handling properties, and esthetic requirements, without compromising bond integrity. All evaluated surface treatments achieved clinically acceptable repair bond strengths. However, high-energy Er:YAG laser treatment and air abrasion are the most effective options for enhancing micromechanical retention and bond strength, whereas low-energy laser treatment should be avoided. The combination of Er:YAG laser treatment at 5 W (250 mJ) with a low-viscosity repair composite produced the highest bond durability after thermocycling. This approach offers a practical and efficient alternative to conventional air-abrasion, particularly when clinicians seek to avoid its operational complexity and messiness while maintaining optimal repair outcomes.

## Data Availability

The original contributions presented in the study are included in the article/Supplementary Material, further inquiries can be directed to the corresponding author.

## References

[1] Bompolaki D Lubisich EB Fugolin AP. Resin-based composites for direct and indirect restorations: clinical applications, recent advances, and future trends. Dent Clin. (2022) 66:517–36. 10.1016/j.cden.2022.05.003 36216444

[2] Amin F Fareed MA Zafar MS Khurshid Z Palma PJ Kumar N. Degradation and stabilization of resin-dentine interfaces in polymeric dental adhesives: an updated review. Coatings. (2022) 12:1094. 10.3390/coatings12081094

[3] Furtado MD Immich F Da Rosa WL Piva E Da Silva AF. Repair of aged restorations made in direct resin composite-A systematic review. Int J Adhes Adhes. (2023) 124:103367. 10.1016/j.ijadhadh.2023.103367

[4] Almutairi MA Salama FS Alzeghaibi LY Albalawi SW Alhawsawi BZ. Surface treatments on repair bond strength of aged resin composites. J Int Soc Prev Commun Dent. (2022) 12:449–55. 10.4103/jispcd.JISPCD_99_22 PMC9615938 36312574

[5] Bourgi R Kharouf N Cuevas-Suárez CE Lukomska-Szymanska M Haikel Y Hardan L. A literature review of adhesive systems in dentistry: key components and their clinical applications. Appl Sci. (2024) 14:8111. 10.3390/app14188111

[6] Cuevas-Suárez CE Nakanishi L Isolan CP Ribeiro JS Moreira AG Piva E. Repair bond strength of bulk-fill resin composite: effect of different adhesive protocols. Dent Mater J. (2020) 39:236–41. 10.4012/dmj.2018-291 31723090

[7] Blum IR Martos R Szalóki M Lynch CD Hegeds C. Effects of different surface treatments and adhesive self-etch functional monomers on the repair of bulk fill composites: a randomised controlled study. J Dent. (2021) 108:103637. 10.1016/j.jdent.2021.103637 33766513

[8] Junior SA Ferracane JL Bona D Á. Influence of surface treatments on the bond strength of repaired resin composite restorative materials. Dent Mater. (2009) 25:442–51. 10.1016/j.dental.2008.09.009 19027938

[9] Florczak-Matyjek A Nikodem A Kensy J Matys J Grzech-Leśniak K. The efficacy of erbium-ion, diode, and CO2 lasers in debonding attachments used during overlay orthodontic treatment and the risk of hard tooth tissue damage compared to traditional methods-an *in vitro* study. Photonics. (2025) 12:621. 10.3390/photonics12060621

[10] Ghavam M Naeemi M Hashemikamangar SS Ebrahimi H Kharazifard MJ. Repair bond strength of composite: effect of surface treatment and type of composite. J Clin Exp Dent. (2018) 10:e520. 10.4317/jced.54030 29930769 PMC6005082

[11] Celiksoz O Yilmaz N Balin E. Effect of ER:YAG laser on repair bond strength of a nano-hybrid composite. J Stomatol. (2022) 75:122–9. 10.5114/jos.2022.117408

[12] Elsahn NA El-Damanhoury HM Elkassas DW. Influence of low-level laser modification and adhesive application mode on the bonding efficiency of universal adhesives to ER:YAG laserablated dentin. J Lasers Med Sci. (2021) 12:e7. 10.34172/jlms.2021.07 34084733 PMC8164904

[13] Saleh RS Safwat EM. The influence of different surface treatment protocols and bonding agents on the repair microtensile bond strength of six-month'aged composite: an *in vitro* study. Braz Dent Sci. (2020) 23:9. 10.14295/bds.2020.v23i4.2119

[14] Dursun MN Ergin E Ozgunaltay G. The effect of different surface preparation methods and various aging periods on microtensile bond strength for composite resin repair. Niger J Clin Pract. (2021) 24:282–91. 10.4103/njcp.njcp_83_20 33605921

[15] Dogan E Cevval Ozkocak BB. The efficacy of er, cr: ySGG laser and contemporary universal adhesive systems on composite resin repair bond strength: an *in vitro* study. Odontology. (2024) 112:1197–208. 10.1007/s10266-024-00932-2 38568323

[16] Ugurlu M Al-Haj Husain N Özcan M. Repair of bulk-fill and nanohybrid resin composites: effect of surface conditioning, adhesive promoters, and long-term aging. Materials (Basel). (2022) 15:4688. 10.3390/ma15134688 35806811 PMC9267362

[17] De Medeiros TC De Lima MR Galvão MR. Repair bond strength of bulk fill composites after different adhesion protocols. J Clin Exp Dent. (2019) 11:e1000. 10.4317/jced.56129 31700573 PMC6825730

[18] Veloso SR Lemos CA Moraes D Do SL Vasconcelos E Pellizzer BC Clinical performance of bulk-fill and conventional resin composite restorations in posterior teeth: a systematic review and meta-analysis. Clin Oral Investig. (2019) 23:221–33. 10.1007/s00784-018-2429-7 29594349

[19] Sengupta A Naka O Mehta SB Banerji S. The clinical performance of bulk-fill versus the incremental layered application of direct resin composite restorations: a systematic review. Evid Based Dent. (2023) 24:143. 10.1038/s41432-023-00905-4 37402908 PMC10516750

[20] Aquino C Mathias C Barreto SC Cavalcanti AN Marchi GM Mathias P. Repair bond strength and leakage of non-aged and aged bulk-fill composite. Oral Health Prev Dent. (2020) 18:a45082. 10.3290/j.ohpd.a45082 32895662 PMC11654506

[21] Şişmanoğlu S Gürcan AT Yıldırım-Bilmez Z Gümüştaş B. Efficacy of different surface treatments and universal adhesives on the microtensile bond strength of bulk-fill composite repair. J Adhes Sci Technol. (2020) 34:1115–27. 10.1080/01694243.2019.1698202

[22] Binhasan M Althobaiti F Alyami R Aljabri K Alabbas T Barakah H. Effect of surface treatments on repair bond strength of aged bulk-fill resin composites. Polymers (Basel). (2025) 17:2326. 10.3390/polym17172326 40942244 PMC12431073

[23] Akgül S Kedici Alp C Bala O. Repair potential of a bulkfill resin composite: effect of different surfacetreatment protocols. Eur J Oral Sci. (2021) 129:e12814. 10.1111/eos.12814 34309074

[24] Yldrm-Ik H Büyükgöze-Dindar M. Influence of different adhesives and surface treatments on shear and tensile bond strength and microleakage with micro-CT of repaired bulk-fill composites. Polymers (Basel). (2025) 17:1680. 10.3390/polym17121680 40574209 PMC12196890

[25] Atalay C Yazici AR Ozgunaltay G. Bond strengths of bulk-fill resin composite repairs: effect of different surface treatment protocols *in vitro*. J Adhes Sci Technol. (2018) 32:921–30. 10.1080/01694243.2017.1395162

[26] Ayar MK Guven ME Burduroglu HD Erdemir F. Repair of aged bulkfill composite with posterior composite: effect of different surface treatments. J Esthet Restor Dent. (2019) 31:246–52. 10.1111/jerd.12391 30194910

[27] Merwade S. The role of oxygen inhibited layer on the shear bond strength of composites-an invitro evaluation. J Conserv Dent Endod. (2007) 10:1–4. 10.4103/0972-0707.42273

[28] Hasnain MS Ahmad SA Chaudhary N Minhaj MA Nayak AK. Degradation and failure of dental composite materials. In: Ahmed W Ali N, eds. Applications of Nanocomposite Materials in Dentistry. Cambridge: Woodhead Publishing (2019). p. 107–21. 10.1016/B978-0-12-813742-0.00006-7

[29] Szczesio-Wlodarczyk A Kopacz K Ranoszek-Soliwoda K Sokolowski J Bociong K. Towards the standardization of artificial aging protocols for dental composites: evaluation of proposed methods. J Funct Biomater. (2025) 16:49. 10.3390/jfb16020049 39997583 PMC11856418

[30] Boussès Y Brulat-Bouchard N Bouchard PO Tillier Y. A numerical, theoretical and experimental study of the effect of thermocycling on the matrix-filler interface of dental restorative materials. Dent Mater. (2021) 37:772–82. 10.1016/j.dental.2021.01.010 33608140

[31] Pieniak D Niewczas AM Pikua K Gil L Krzyzak A Przystupa K Effect of hydrothermal factors on the microhardness of bulk-fill and nanohybrid composites. Materials (Basel). (2023) 16:2130. 10.3390/ma16052130 36903245 PMC10004216

[32] Eren MC Çobanoglu N. Immediate and delayed repair of bulk-fill composite resin. Int Dent J. (2024) 74:S133. 10.1016/j.identj.2024.07.981

[33] Bektas ÖÖ Eren D Siso SH Akin GE. Effect of thermocycling on the bond strength of composite resin to bur and laser treated composite resin. Lasers Med Sci. (2012) 27:723–8. 10.1007/s10103-011-0958-2 21833556

[34] Rashidi M Berangi S Chiniforush N Ahmadi E Omrani LR. Microtensile repair bond strength of a composite after accelerated artificial aging: effect of the air abrasion, bur, ER:YAG laser, two-step self-etch bonding, and universal bonding repair system. J Lasers Med Sci. (2022) 13:e18. 10.34172/jlms.2022.18 35996482 PMC9392884

[35] Kimyai S Mohammadi N Navimipour EJ Rikhtegaran S. Comparison of the effect of three mechanical surface treatments on the repair bond strength of a laboratory composite. Photomed Laser Surg. (2010) 28:S-25. 10.1089/pho.2009.2598 20950189

[36] Hasan N. The influence of ER:YAG laser, alumi-num oxide and diamond bur on surface treatment of aged composite resin to repair restoration. Al-Rafidain Dental Journal. (2012) 12:257–65. 10.33899/rden.2012.65076

[37] Kiomarsi N Saburian P Chiniforush N Karazifard MJ Hashemikamangar SS. Effect of thermocycling and surface treatment on repair bond strength of composite. J Clin Exp Dent. (2017) 9:e945. 10.4317/jced.53721 28936282 PMC5601109

[38] Barcellos DC Santos VM Niu LN Pashley DH Tay FR Pucci CR. Repair of composites: effect of laser and different surface treatments. Int J Adhes Adhes. (2015) 59:1–6. 10.1016/j.ijadhadh.2015.01.008

[39] Cho SD Rajitrangson P Matis BA Platt JA. Effect of ER, CR: ySGG laser, air abrasion, and silane application on repaired shear bond strength of composites. Oper Dent. (2013) 38:58–6640. 10.2341/11-054-L 23131133

[40] Oskoee PA Mohammadi N Chaharom ME Kimyai S Azar FP Rikhtegaran S Effect of surface treatment with ER; CR: ySSG, nd: yAG, and CO_2_ lasers on repair shear bond strength of a silorane-based composite resin. J Dent Res. (2013) 7:61. 10.5681/joddd.2013.011 PMC3713862 23875082

[41] Hatipoglu M Barutcigil C. Effects of erbiumand chromiumdoped yttrium scandium gallium garnet and diode lasers on the surfaces of restorative dental materials: a scanning electron microscope study. Niger J Clin Pract. (2015) 18:213–20. 10.4103/1119-3077.151044 25665995

[42] Duran I Ural Ç Yilmaz B Tatar N. Effects of ER:YAG laser pretreatment with different energy levels on bond strength of repairing composite materials. Photomed Laser Surg. (2015) 33:320–5. 10.1089/pho.2014.3859 26067940

[43] Teixeira EC Bayne SC Thompson JY Ritter AV Swift EJ. Shear bond strength of self-etching bonding systems in combination with various composites used for repairing aged composites. J Adhes Dent. (2005) 7:159. 16052765

[44] Tugut F Akin H Mutaf B Akin GE Ozdemir AK. Strength of the bond between a silicone lining material and denture resin after ER:YAG laser treatments with different pulse durations and levels of energy. Lasers Med Sci. (2012) 27:281–5. 10.1007/s10103-010-0862-1 21153674

[45] Batista GR Kamozaki MB Gutierrez NC Ferraz Caneppele TM Torres G Effects of different surface treatments on composite repairs. J Adhes Dent. (2015) 17:421. 10.3290/j.jad.a35013 26525006

[46] Rossato DM Bandeca MC Saade EG Influence of ER:YAG laser on surface treatment of aged composite resin to repair restoration. Laser Phys. (2009) 19:2144–9. 10.1134/S1054660X09210105

[47] Sirin Karaarslan E Ozsevik AS Cebe MA Surmelioglu G Tosun HD Yildiz S Bond strength of repaired composite resins: surface treatments, adhesive systems, and composite type. J Adhes Sci Technol. (2016) 30:520–33. 10.1080/01694243.2015.1111187

[48] Ahlholm P Staxrud F Sipilä K Vallittu P. Repair bond strength of bulk-fill composites: influence of different primers and direction of debonding stress. Biomater Investig Dent. (2023) 10:2258924. 10.1080/26415275.2023.2258924 37753305 PMC10519262

[49] Ahmadizenouz G Esmaeili B Taghvaei A Jamali Z Jafari T Daneshvar FA Effect of different surface treatments on the shear bond strength of nanofilled composite repairs. J Dent Res. (2016) 10:9. 10.15171/joddd.2016.002 PMC4831615 27092209

[50] Eren D Doan CA Bekta ÖÖ. Effect of different surface treatments and roughness on the repair bond strength of aged nanohybrid composite. Photobiomodulation, Photomedicine, and Laser Surgery. (2019) 37:473–82. 10.1089/photob.2018.4585 31081715

[51] Tsutsumi MS De Souza TF De S Batista VE De A Matuda LS Bond strength repair of a bulk-fill composite using different adhesive systems and resin composites. Res Soc Dev. (2021) 10:e31410514951. 10.33448/rsd-v10i5.14951

[52] Benzi JG Pucci CR Freitas MR Liporoni S Zanatta PC RF. Bonding performance for repairs using bulk fill and conventional methacrylate composites. Int J Dent. (2021) 2021:2935507. 10.1155/2021/2935507 34956366 PMC8702360

